# Supplementary material for: R-loop induced G-quadruplex in non-template promotes transcription by successive R-loop formation
Source: Nat Commun. 2020 Jul 7;11:3392. doi: 10.1038/s41467-020-17176-7 (PMC7341879; doi:10.1038/s41467-020-17176-7)
Supplement: Supplementary file 1 — Supplementary Information [file 41467_2020_17176_MOESM1_ESM.pdf]

SUPPLEMENTARY INFORMATION

R-loop induced G-quadruplex in non-template promotes transcription by successive R-loop formation

Chun-Ying Lee, Christina McNerney, Kevin Ma, Walter Zhao, Ashley Wang and Sua Myong

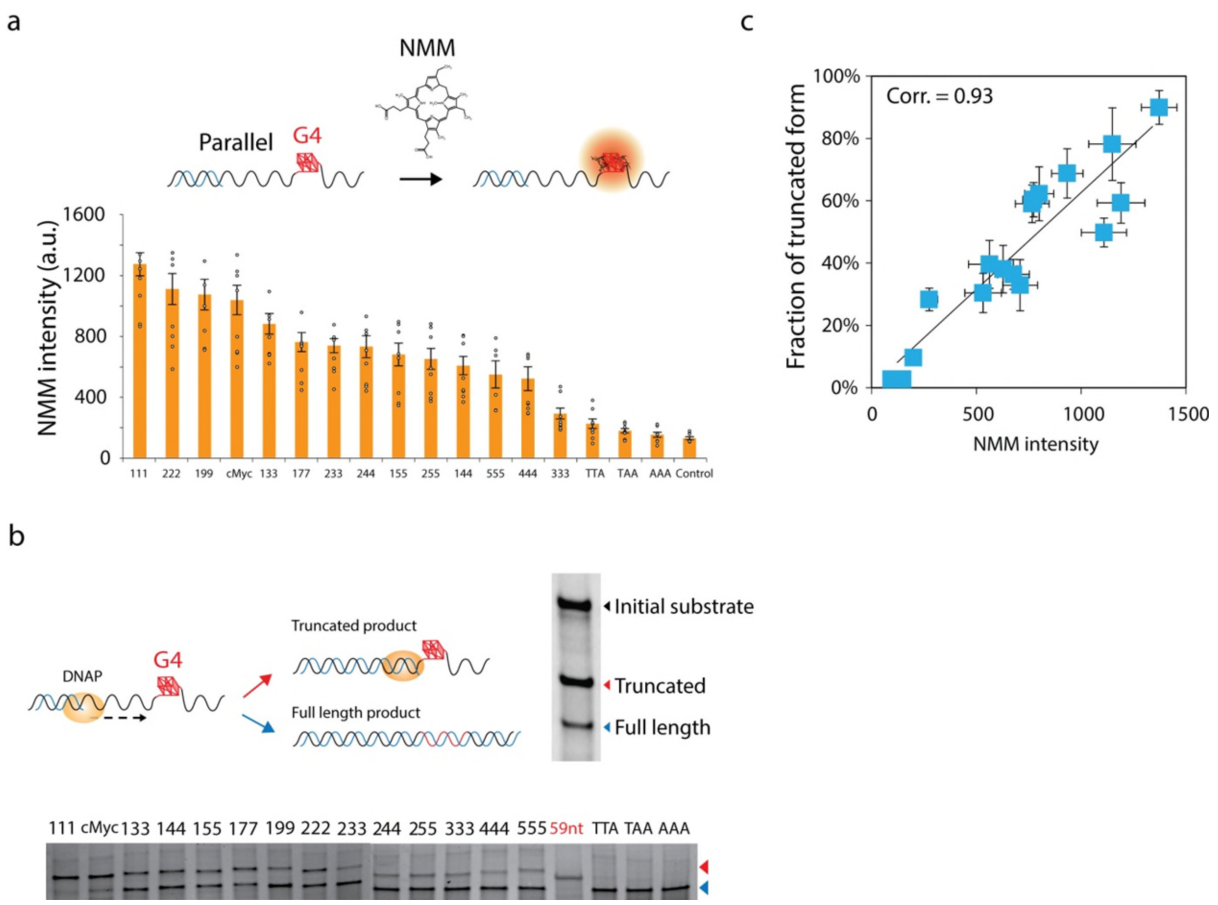

Supplementary Fig. 1: Parallel G4 folding propensity is highly correlated to its biophysical stability.

a, Parallel G4 formation is determined by the increase of fluorescence upon the binding of N-Methyl mesoporphyrin IX (NMM). 17 PQS sequences (**Supplementary Table 2**) are tested and sorted by NMM intensity. Data are presented as mean values ± SEM of n = 8 independent experiments. **b,** Biological barrier effect due to G4 folding and structural stability is measured by DNA polymerase stop assay. The stronger G4 inhibits DNA extension and leads more truncated product; in contrast, the weaker G4 is unfolded by DNA polymerase and produces full-length dsDNA. Shown is a single presentative image from one of n = 4 independent experiments. Full scan images with molecular markers are provided in Source Data file. **c,** Data from **a** and **b** are plotted to show the correlation with format mean values ± SEM. The fraction of truncated product is highly correlated to NMM intensity, indicating the propensity of parallel G4 folding may be responsible to the barrier effect in biological process. Pearson's correlation coefficient is indicated. Data is provided in supplementary table 3.14.

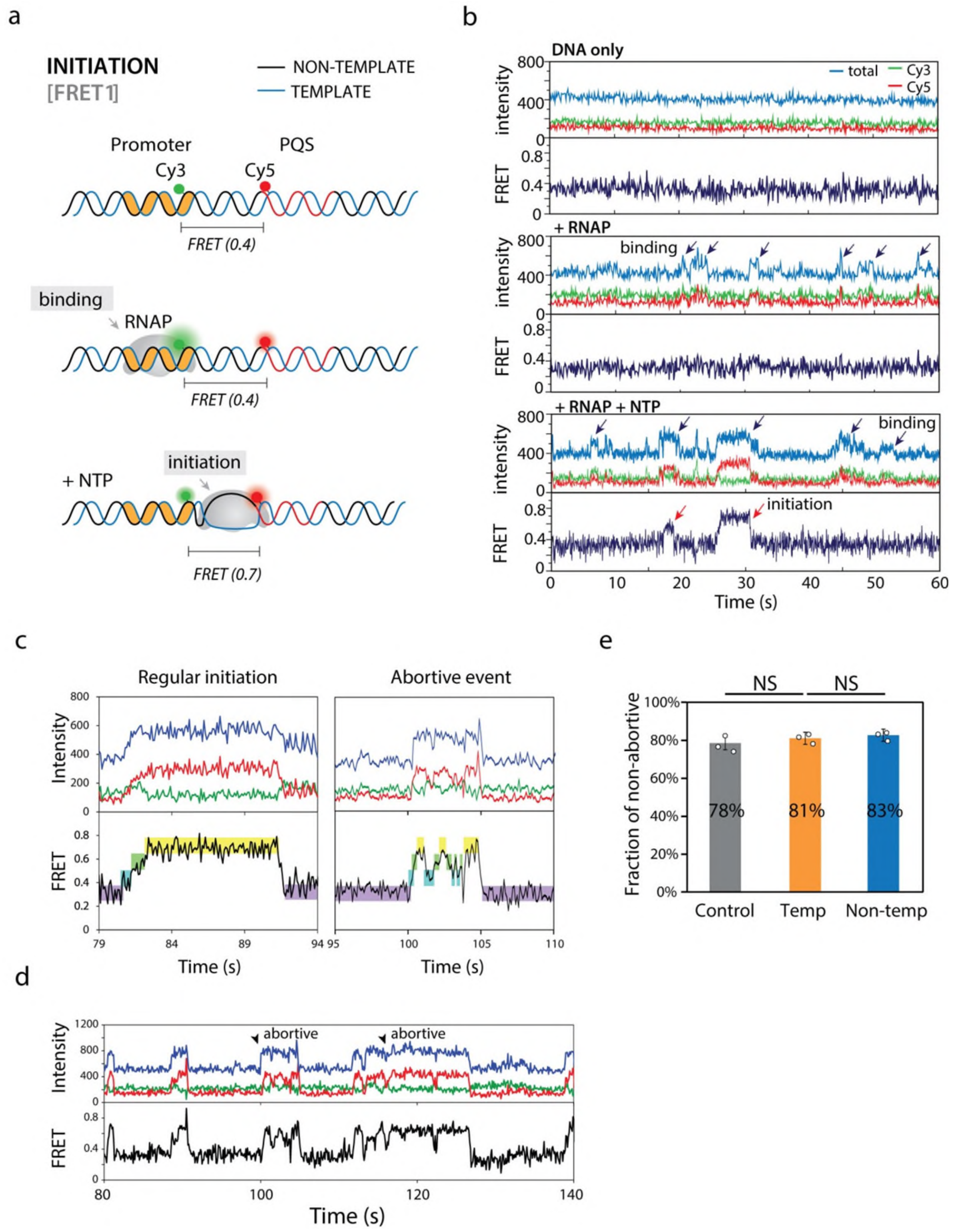

Supplementary Fig. 2: FRET1 construct can be used to identify binding, initiation and abortive initiation.

a, Scheme of FRET1 proposes the FRET transitions to binding (0.4) and initiation (0.7). Promoter, PQS and RNAP are colored in orange, red, and gray. **b,** Trace examples demonstrates the

difference between binding and initiation in FRET1. Addition of RNAP causes Cy3 PIFE signal (blue arrow) but no FRET burst (red arrow), indicating this is binding signal. Addition of RNAP and NTP together cause PIFE and FRET burst, indicating the structural change due to opening of transition bubble. **c,** FRET pattern example shows the abortive event is identified by repeated transition between high FRET and mid FRET. **d,** Both regular initiation and abortive initiation can be observed in one single trace, indicating the randomness of each event. **e,** The fraction of regular initiation is quantified by the count of regular initiation from all the events. All three test sequences show no significant difference and about 80% regular initiation occurs during the first stage of transcription. Data are presented as mean ± SEM of n = 3 independent experiments. Statistics analysis performed by one-sided Pearson's chi square test shows no significance (NS). Exact *P*-values are provided in supplementary table 3.15.

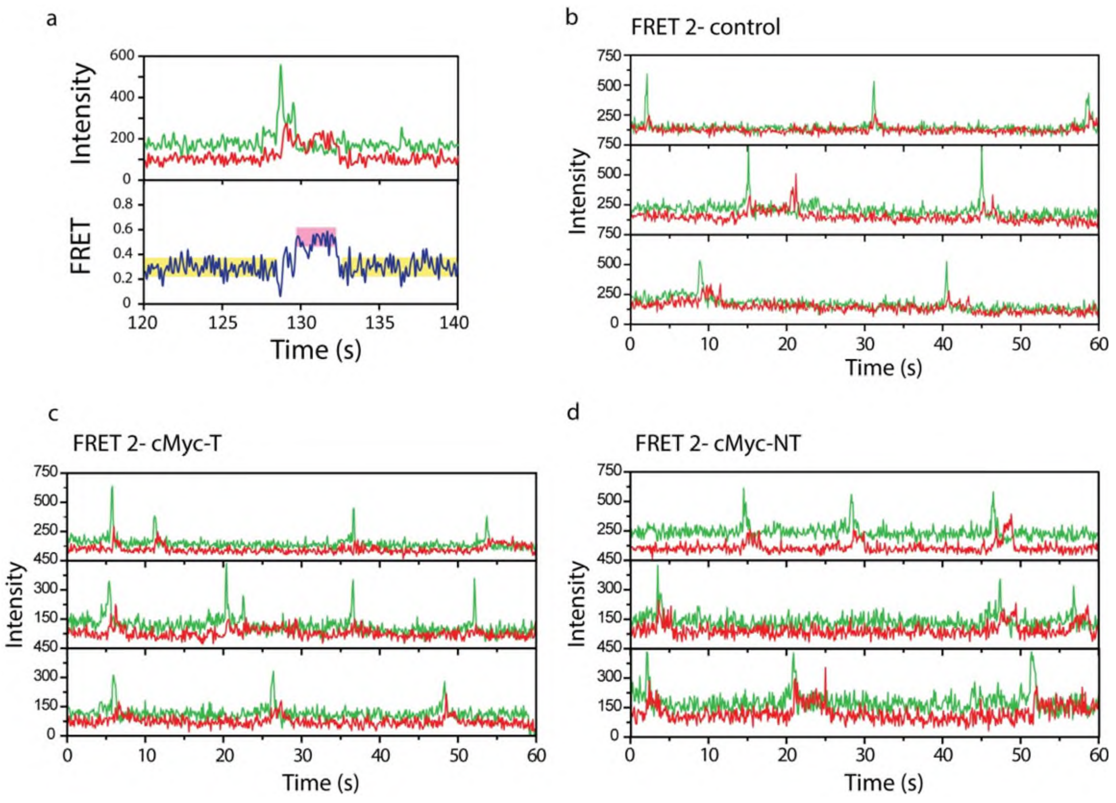

Supplementary Fig. 3: FRET2 construct has same transcription pattern for control, template and non-template.

a, Transcription pattern of FRET2. The pattern starts with a Cy3 PIFE, followed by a short transition and a Cy5 PIFE, and then it transits back to original FRET state. **b,c,d,** The trace examples of control, template and non-template, respectively.

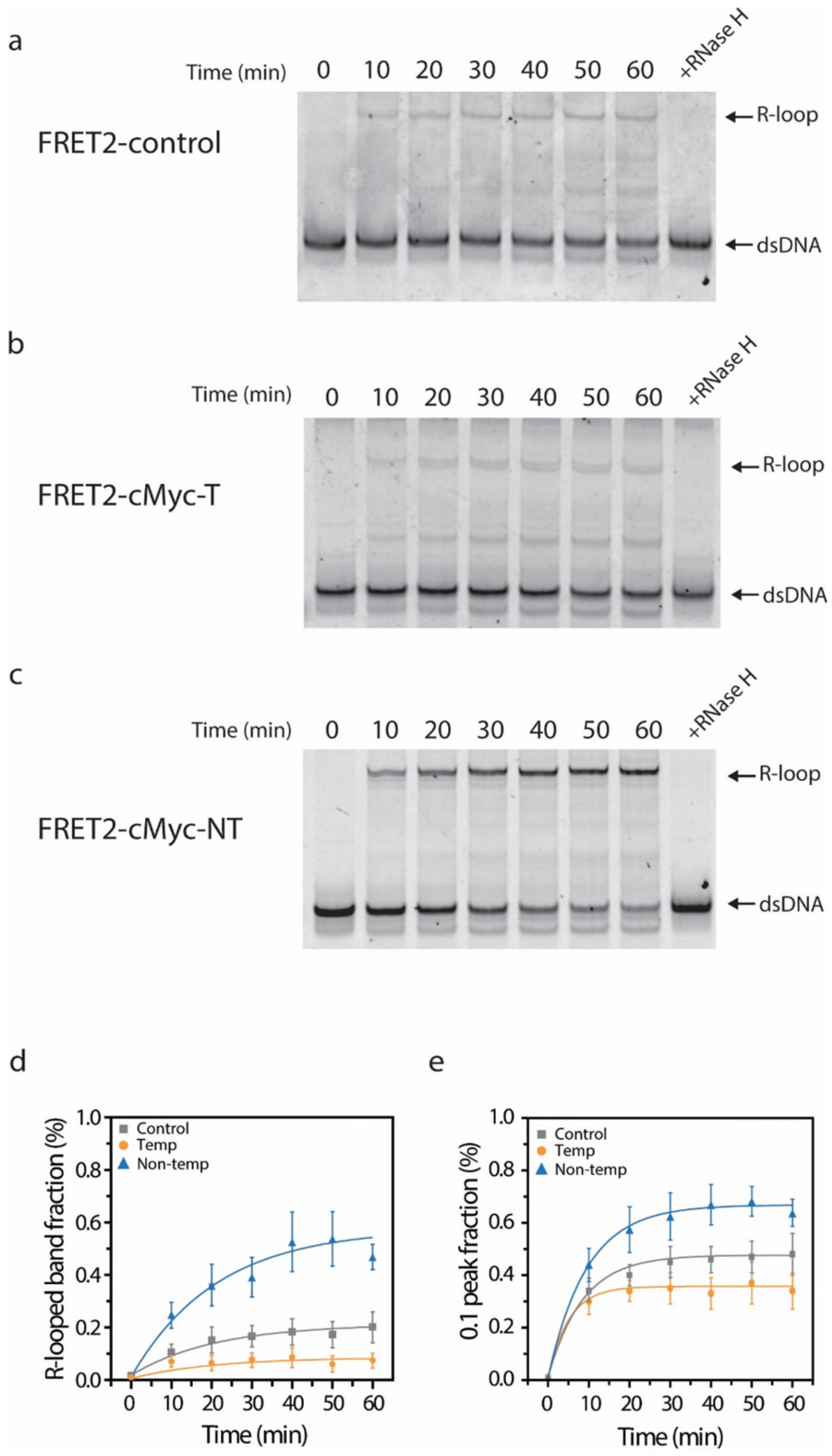

Supplementary Fig. 4: Example of EMSA gel for control, template and non-template and the plot of fraction.

a,b,c, The dsDNA and R-looped state are distinguished by 10% PAGE gel. **d,** The real time fration of 0.1 peak quanitified from FRET histogram in **Fig. 5d**. **e,** The real time fraction of R-loop band quantified from **a,b,c** by ImageJ software. The correlation of R-loop and 0.1peak is plotted in **Fig.**

5g. For **a**, **b**, **c**, each images is shown as one representative images of n = 3 independent experiments and is taken under Cy5 channel. Source data of full scan image and molecular marker are stained separately and provided as Source Data file. For **d**, data are presented as mean ± SEM from the quantification of **a**, **b** and **c**, provided in supplementary table 3.16. For **e**, data are presented as mean ± SEM of n = 5 independent experiment. Data are plotted from the quantification of FRET histogram in **Fig. 5d** and provided in supplementary table 3.17.

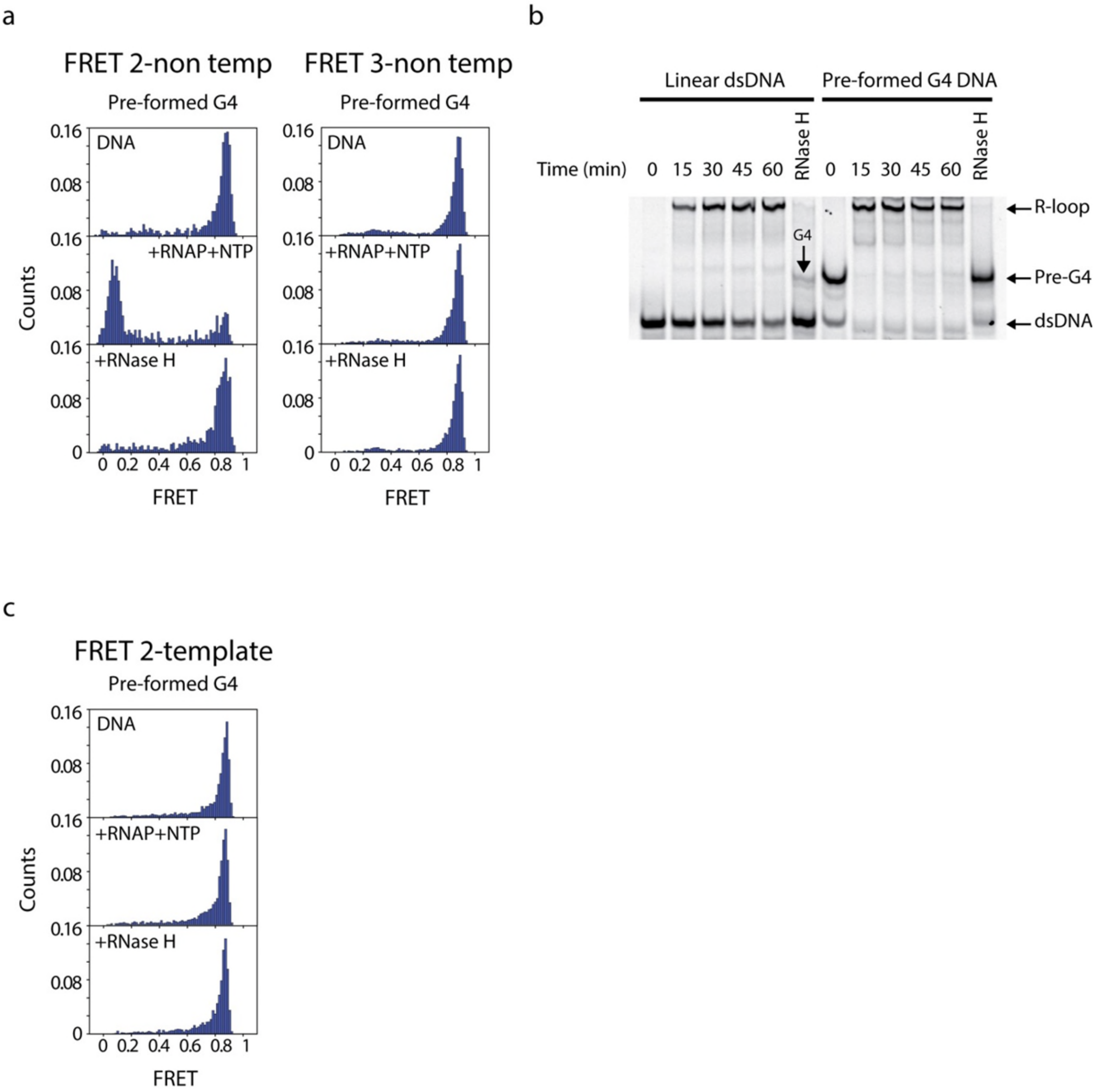

Supplementary Fig. 5: Pre-formed G4-NT construct forms G4/R-loop immediately in the transcription.

a, Pre-formed G4-NT DNA of FRET2 and FRET3 construct show different transitions during transcription. It is due to the labeling site where Cy5 in FRET3 is more sensitive to G4 structure and remains high FRET value. Both constructs have one 0.9 FRET peak after RNase H digestion, indicating that G4 structure is remained during transcription process. **b,** Pre-formed G4 DNA has faster transition from DNA state to R-looped state, and G4 structure remains the same state after RNase H digestion. Shown is a single representative image from n = 2 independent experiments and is taken under Cy5 emission channel. Source data of full scan and molecular marker is stained separately and provided as Source Data file. **c,** Pre-formed G4 at template strand remains high FRET after initiation transcription, indicating no R-loop forms during transcription. In other words, pre-formed PQS-T blocks transcription.

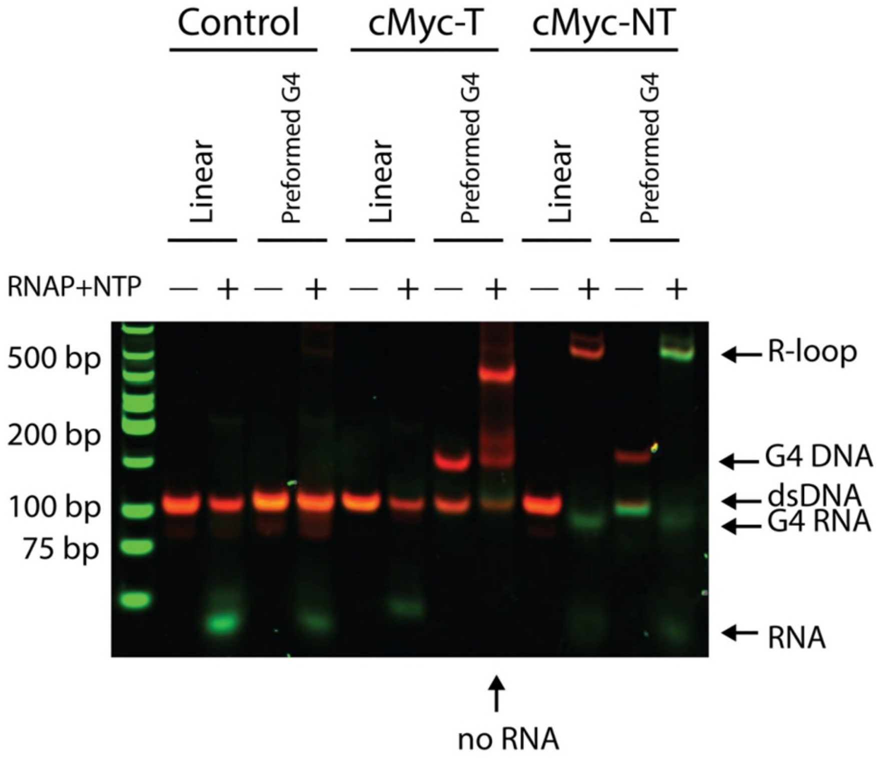

Supplementary Fig. 6: Pre-formed G4-T construct blocks transcription.

This gel compares the difference between linear and pre-formed constructs on transcription. For control DNA, there is only one band for both linear and pre-formed G4 samples, indicating it only forms dsDNA. For template and non-template, pre-formed DNA samples have one addition upper band which is the G4 DNA. Interestingly, among all these constructs, only pre-formed template G4 has no RNA product, indicating the G4 in template strand blocks the RNAP and inhibits transcription process. Noted that, additional dsDNA band in pre-formed G4 is due to the annealing process, which is difficult to reach 100% pre-formed G4. Shown is a single representative image from n = 3 independent experiments.

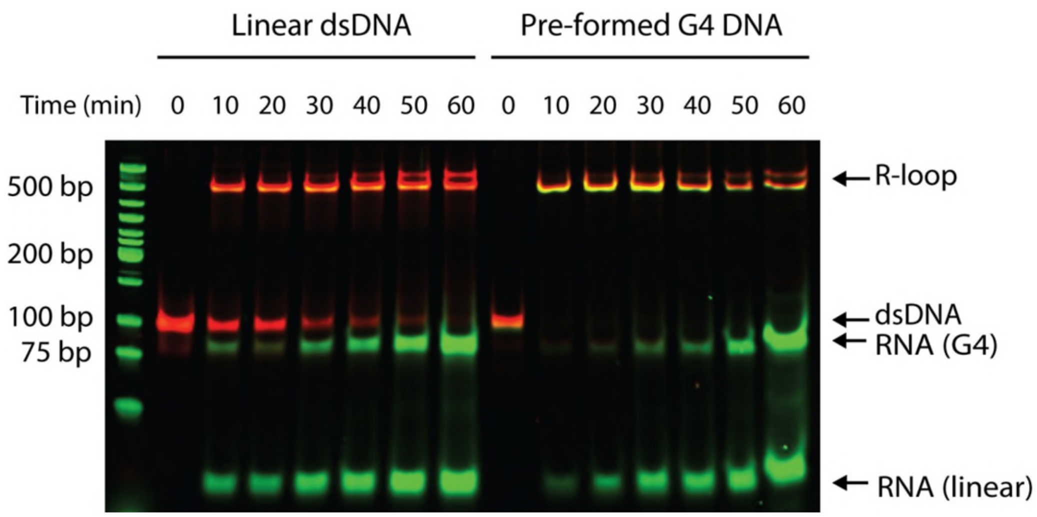

Supplementary Fig. 7: Pre-formed G4 DNA is still able to produce RNA.

The gel compares linear and pre-formed G4 non-template DNA. Both constructs show the bands shift upward (yellow), indicating the R-loop formation. RNA stained by Sybr-Green II are colored in green. Shown is a single representative image from n = 3 independent experiments.

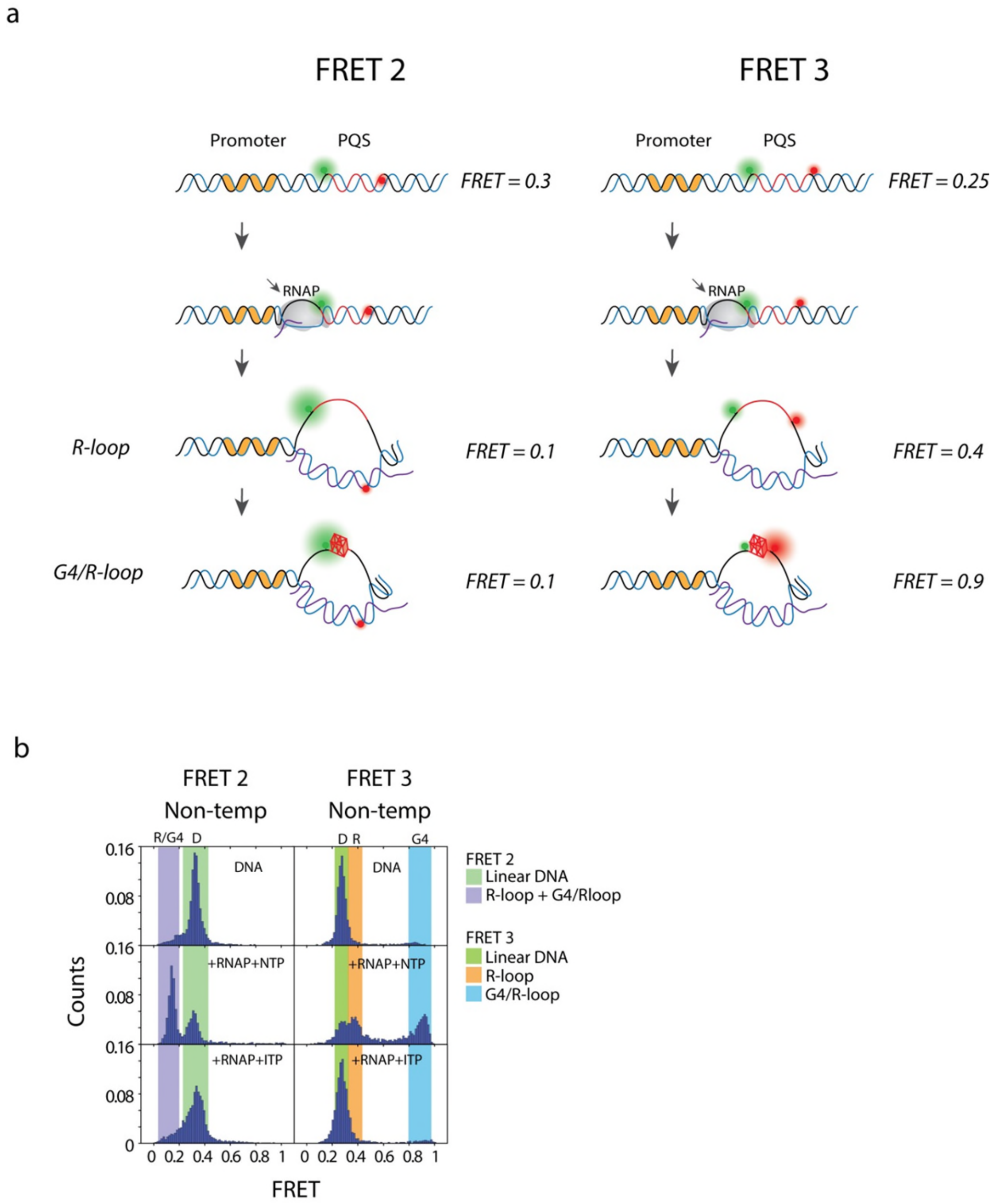

Supplementary Fig. 8: FRET states and corresponding DNA structure. a, FRET2 construct has Cy3 and Cy5 labeled at each strand, which is unable to recognize R-loop and G4/R-loop state. In FRET3, both dyes are labeled in the non-template strand across PQS region. It enables to distinguish the two R-loop states. **b,** FRET state of R-looped structure can be distinguished by ITP substitution experiment. As shown in **Fig. 7g**, ITP diminished R-loop states as well as G4 structure. When applied ITP mix, 0.1 peak of FRET2 and 0.4 and 0.9 peak of FRET3 disappear, indicating those peaks are corresponding to R-loop.

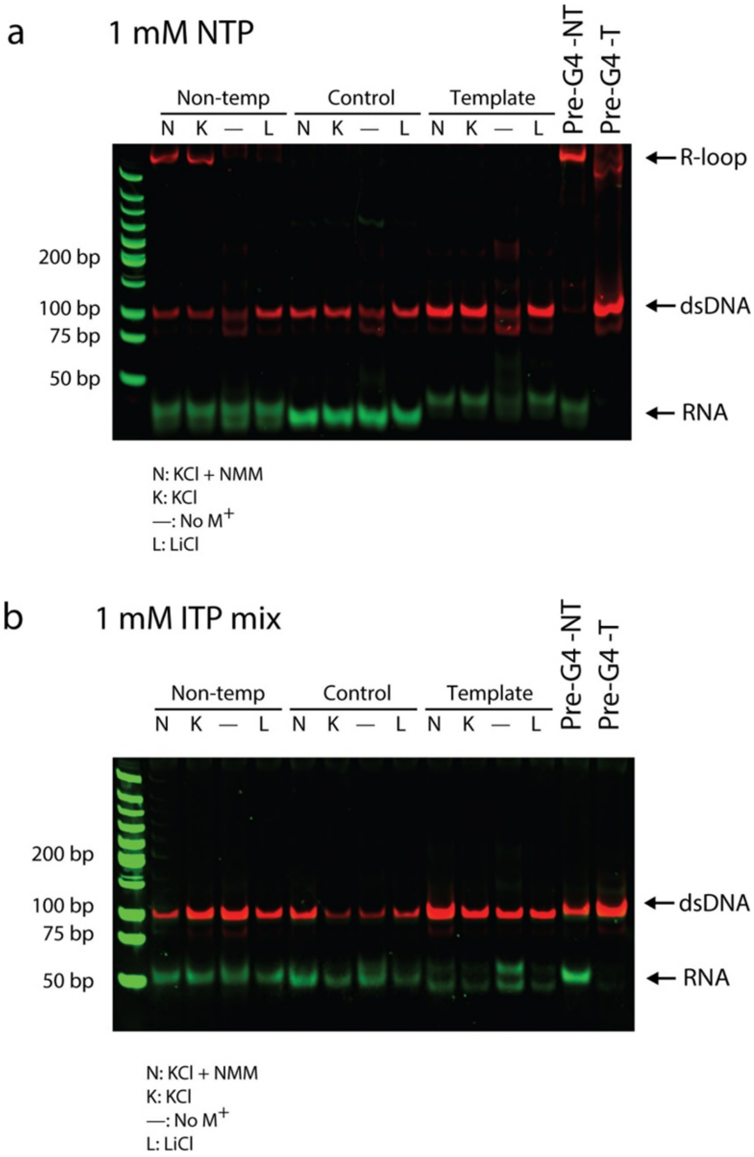

Supplementary Fig. 9: G4 stabilizing buffer test with NTP and ITP mix.

These example gels compare four buffer conditions, N: 50 mM KCl + 1 μM NMM; K: 50 mM KCl; —: No monovalent ion; L: 50 mM LiCl. Two more pre-formed G4 constructs were tested in 50 mM KCl. DNA substrate was labeled with Cy5 dye, showing in red, and ladder and RNA were stained by Sybr-Green II, showing in green. **a**, In the presence of 1 mM NTP mix. **b**, In the presence of 1 mM ITP mix. The gel shows R-loop forms only in NTP condition. Also, pre-formed G4-template has no RNA product. Due to the fluorescence enhancement effect of pre-formed G4, the samples were diluted 5 folds before sample loading. Each image is one representative image from n = 3 independent experiments. Data are quantified (**Supplementary table 3-11, 12, 13**) and plotted as bar graph to show the correlation of R-loop and RNA production (**Fig. 8b-d**).

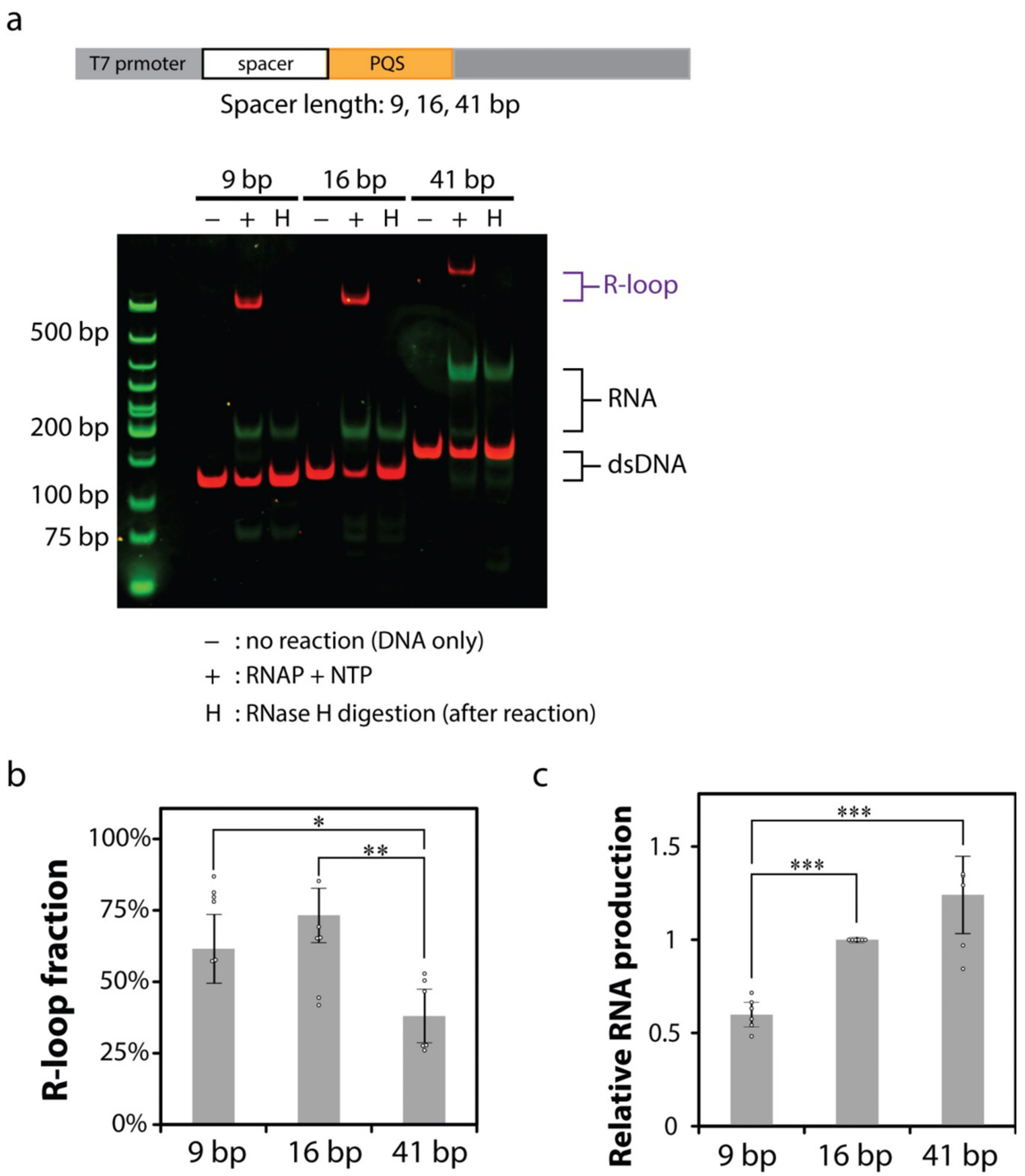

Supplementary Fig. 10: R-loop formation and RNA production depend on PQS position. **a,** Transcription with different PQS position was tested by inserting varying length of spacer between T7 promoter and PQS. Each construct was initiated reaction and further digested by RNase H. Shown is a single representative image from n = 6 independent experiments. Data is quantified and provided in supplementary table 3.18 (for **b**) and 3.19 (for **c**). **b,** The R-loop fraction was quantified by Cy5 intensity in the same lane. **c,** RNA production was quantified by green fluorescence intensity (stained by SybrGreen II) and normalized to DNA band (Cy5 intensity) and

16 bp production. For **b**, **c**, data are presented as mean ± SEM of n = 6 independent

experiments, quantified from **a**. Statistical analysis, $^*P < 0.05$, $^{**}P < 0.005$, $^{***}P < 0.0005$, is reported by two-sided unpaired t-test.

Supplementary Table 1: Sequences of DNA substrate in each assay.

**Item name**	**Sequence (5’→ 3’)**	**Substrate**
Clone sequence	CTCGAGGCGAATTAATACGACTCACTATAGGGGAATTGT GAGCGGATAACAATTCCCCTCTAGAGAATTCTTT-**PQS**-T TACCGGTTTTGGTTGAAGGTAGTGGTAGTGGTGATATC CTGCAGGAAAGGAGAGTAGCAATGGGTACC	Plasmid cloning
Biotin forward primer	Biotin-GCTTTCGTCTTCACCTCGAG	PCR primer
T7 terminator reverse primer	AAACCCCTCCGTTTAGAGAGGGGTTATGCTAGTTATTTG TAGAGCTCATCCATGCC	PCR primer
Beacon probe	Cy3-TTCACCCTCTCCACGGAC-BHQ_2	Ensemble, smBeacon
T7 promoter top	GAAATTAATACGACTCACTATA	Termination
T7 promoter bottom	TATAGTGAGTCGTATTAATTTC	Termination
Biotin-18 mer	GCCTCGCTGCCGTCGCCA-Biotin	
FRET1-control-Cy3	TGGCGACGGCAGCGAGGCTAAATTAATACGACTCAC/Cy 3/ATAGGGAGACCACAACGTTAGGCGACGGCAGCGAGG TTATCAGCTCCAGGTCT	Binding, Initiation, Dual PIFE
FRET1-control-Cy5	AGACCTGGAGCTGATAACCTCGCTGCCGTCGCC/Cy5/A ACGTTGTGGTCTCCCTATAGTGAGTCGTATTAATTTA	Binding, Initiation, Dual PIFE
FRET1-CMT-Cy3	TGGCGACGGCAGCGAGGCTAAATTAATACGACTCAC/Cy 3/ATAGGGAGACCACAACGTTACCCACCCTACCCACCCT TATCAGCTCCAGGTCT	Binding, Initiation, Dual PIFE
FRET1-CMT-Cy5	AGACCTGGAGCTGATAAGGGTGGGTAGGGTGGG/Cy5/A ACGTTGTGGTCTCCCTATAGTGAGTCGTATTAATTTA	Binding, Initiation, Dual PIFE
FRET1-CMNT-Cy3	TGGCGACGGCAGCGAGGCTAAATTAATACGACTCAC/Cy 3/ATAGGGAGACCACAACGTTAGGGTGGGTAGGGTGGG TTATCAGCTCCAGGTCT	Binding, Initiation, Dual PIFE
FRET1-CMNT-Cy5	AGACCTGGAGCTGATAACCCACCCTACCCACCC/Cy5/A ACGTTGTGGTCTCCCTATAGTGAGTCGTATTAATTTA	Binding, Initiation, Dual PIFE
FRET2-control-Cy3	TGGCGACGGCAGCGAGGCTAAATTAATACGACTCACTA TAGGGAGACCACAACG/Cy3/TAGGCGACGGCAGCGAG GTTATCAGCTCCAGGTCT	Elongation
FRET2-control-Cy5	AGACCTGGAGCTGA/Cy5/AACCTCGCTGCCGTCGCCTA	Elongation,

	ACGTTGTGGTCTCCCTATAGTGAGTCGTATTAATTTA	Dual PIFE
FRET2-CMT-Cy3	TGGCGACGGCAGCGAGGCTAAATTAATACGACTCACTA TAGGGAGACCACAACG/Cy3/TACCCACCCTACCCACCC TTATCAGCTCCAGGTCT	Elongation
FRET2-CMT-Cy5	AGACCTGGAGCTGA/Cy5/AAGGGTGGGTAGGGTGGGT AACGTTGTGGTCTCCCTATAGTGAGTCGTATTAATTTA	Elongation, Dual PIFE
FRET2-CMNT-Cy3	TGGCGACGGCAGCGAGGCTAAATTAATACGACTCACTA TAGGGAGACCACAACG/Cy3/TAGGGTGGGTAGGGTGG GTTATCAGCTCCAGGTCT	Elongation
FRET2-CMNT-Cy5	AGACCTGGAGCTGA/Cy5/AACCCACCCTACCCACCCTA ACGTTGTGGTCTCCCTATAGTGAGTCGTATTAATTTA	Elongation, Dual PIFE
FRET3-CMNT-top	TGGCGACGGCAGCGAGGCTAAATTAATACGACTCACTA TAGGGAGACCACAACG/iCy3/TAGGGTGGGTAGGGTGG GT/Cy5/ATCAGCTCCAGGTCT	G4 formation
FRET3-CMNT-bottom	AGACCTGGAGCTGATAACCCACCCTACCCACCCAACGT TGTGGTCTCCCTATAGTGAGTCGTATTAATTTA	G4 formation
FRET3-control-top	TGGCGACGGCAGCGAGGCTAAATTAATACGACTCACTA TAGGGAGACCACAACGT/iCy3/TAGGCGACGGCAGCGA GGT/Cy5/ATCAGCTCCAGGTCT	G4 formation
FRET3-control-bottom	AGACCTGGAGCTGATAACCTCGCTGCCGTCGCCTAAC GTTGTGTCTCCCTATAGTGAGTCGTATTAATTTA	G4 formation

Supplementary Table 2: PQS sequences.

**PQS name**	**Sequence (5'→ 3')**
111	GGG T GGG T GGG T GGG
cMyc	GGG T GGG TA GGG T GGG
133	GGG T GGG TTT GGG TTT GGG
144	GGG T GGG TTTT GGG TTTT GGG
155	GGG T GGG TTTTT GGG TTTTT GGG
177	GGG T GGG TTTTTTT GGG TTTTTTT GGG
199	GGG T GGG TTTTTTTTT GGG TTTTTTTTT GGG
222	GGG TT GGG TT GGG TT GGG
233	GGG TT GGG TTT GGG TTT GGG
244	GGG TT GGG TTTT GGG TTTT GGG
255	GGG TT GGG TTTTT GGG TTTTT GGG
333	GGG TTT GGG TTT GGG TTT GGG
444	GGG TTTT GGG TTTT GGG TTTT GGG
555	GGG TTTTT GGG TTTTT GGG TTTTT GGG
TTA	GGG TTA GGG TTA GGG TTA GGG
TAA	GGG TAA GGG TAA GGG TAA GGG
AAA	GGG AAA GGG AAA GGG AAA GGG
Control	GTCTGTACTGAGTCAACTAACGACAAG

Supplementary Table 3: Statistics results

Supplementary Table 3.1: Normalized rate of Cy3 increase (Fig. 1e)

Name	Independent experiments						Mean rate	SEM	*P*-value Control	*P*-value Template
Control	1.000	1.000	1.000	1.000	1.000	1.000	1.000	0.030		
111-T	0.806	0.894	0.997	0.957	0.942	0.982	0.930	0.026	0.013	
cMyc-T	1.016	1.193	1.148	0.954	0.992	1.105	1.068	0.035	0.060	
144-T	0.882	0.996	0.987	0.994	0.949	0.946	0.959	0.016	0.019	
222-T	0.807	0.959	0.732	0.815	0.854	0.805	0.829	0.028	2.53E-05	
199-T	0.982	1.014	1.099	0.965	0.941	0.975	0.996	0.021	0.842	
333-T	0.906	1.072	1.227	0.985	0.975	0.915	1.013	0.045	0.751	
TTA-T	0.802	1.000	1.047	1.040	1.031	0.691	0.935	0.056	0.237	
555-T	0.850	0.985	1.032	1.107	1.058	0.954	0.998	0.034	0.940	
AAA-T	0.920	0.911	1.044	0.975	0.943	0.921	0.952	0.019	0.018	
111-NT	1.093	0.961	1.155	1.211	1.113	1.541	1.179	0.073	0.021	4.57E-03
cMyc-NT	1.218	1.252	1.237	1.176	1.553	1.754	1.365	0.087	6.98E-04	5.15E-03
144-NT	1.198	1.184	1.182	1.302	1.251	1.175	1.216	0.019	3.40E-08	1.12E-07
222-NT	1.245	1.386	1.534	1.254	1.246	1.155	1.303	0.050	2.92E-05	1.25E-06
199-NT	0.970	1.195	1.259	1.251	1.974	1.138	1.298	0.130	0.004	5.26E-03
333-NT	1.229	1.027	1.352	1.391	1.418	1.312	1.288	0.054	8.41E-05	1.13E-03
TTA-NT	1.068	1.228	1.201	1.155	1.154	1.154	1.160	0.020	1.97E-06	1.58E-03
555-NT	1.156	1.072	1.270	1.282	1.230	1.585	1.266	0.065	8.51E-04	1.92E-03
AAA-NT	1.218	1.001	1.186	1.311	1.195	1.254	1.194	0.039	1.74E-04	6.15E-05

* SEM is presented as 95% confidence limit.

* *P*-values are reported by two-sided unpaired t-test.

Supplementary Table 3.2: Cy3 burst rate (events per min) (Fig. 1h)

Name	Mean rate	SEM	N value	*P*-value Control	*P*-value Template
Control	1.32	0.24	164		
Template	1.20	0.18	222	0.199	
Non-template	1.81	0.16	475	9.59E-03	8.97E-03

* Data process was described in method. N value means the number of independent events. Raw data are provided in Source Data file. .

* SEM is presented as 95% confidence limit.

* *P*-values are reported by Kruskal-Wallis test (one sided).

Supplementary Table 3.3: Rate of binding events (/sec) (Fig. 3c)

[RNAP] (nM)	Mean rate	SEM	N value
10	0.044	0.008	209
50	0.064	0.006	479
100	0.090	0.003	438

* Data process was described in method. N value means the number of independent events. Raw data are provided in Source Data file.

* SEM is presented as 95% confidence limit.

Supplementary Table 3.4: Average number of binding events per trace (Fig. 3d)

				P-value	
Name	Mean	SEM	N value	Control	Template
Control	3.33	0.31	190		
Template	3.40	0.30	154	0.761	
Non-template	3.64	0.31	155	0.212	0.337

* Data process was described in method. N value means the number of independent events. Raw data are provided in Source Data file.

* SEM is presented as 95% confidence limit.

* *P*-values are reported by two-sided unpaired t-test.

Supplementary Table 3.5: Rate of initiation events (/sec) (Fig. 3g)

[NTP] (μM)	Mean rate	SEM	N value
10	0.020	0.011	40
100	0.045	0.013	470
1000	0.083	0.009	479

* Data process was described in method. N value means the number of independent events. Raw data are provided in Source Data file.

* SEM is presented as 95% confidence limit.

Supplementary Table 3.6: Fraction of initiation complex (Fig. 3h)

Name	Mean	SEM	N value	*P*-value	
				Control	Template
Control	95%	2%	573		
Template	96%	1%	611	0.170	
Non-template	96%	2%	575	0.396	0.609

* The data are represented of n = 3 independent measurements. N value means the total number of samples. SEM is determined by binomial distribution with 95% confidence interval.

* *P*-values are reported by Pearson's chi-squared test (one-sided).

Supplementary Table 3.7: Fraction of successful elongation (Fig. 4d)

Name	Mean	SEM	N value	*P*-value	
				Control	Template
Control	52%	2%	1839		
Template	44%	3%	997	1.45E-04	
Non-template	58%	2%	1585	1.86E-04	1.00E-06

* The data are represented of n = 7 independent measurements. N value means the total number of samples. SEM is determined by binomial distribution with 95% confidence interval.

* *P*-values are reported by Pearson's chi-squared test (one-sided).

Supplementary Table 3.8: Rate of transcription events (per min) (Fig. 5c)

Name	Mean rate	SEM	N value	*P*-value	
				Control	Template
Control	3.4	0.3	229		
Template	3.2	0.2	231	0.114	
Non-template	4.0	0.3	209	0.0304	0.0005

* Data process was described in method. N value means the number of independent events. Raw data are provided in Source Data file.

* SEM is presented as 95% confidence limit.

* *P*-values are reported by Kruskal-Wallis test (one-sided).

Supplementary Table 3.9: Fraction of R-loop to RNAP concentration (Fig. 5i)

Name	[RNAP] (μM)	Mean R-loop fraction	SEM
Control	0.1	2%	1%
	0.25	3%	1%
	0.5	5%	0%
	1	7%	1%
Template	0.1	0%	0%
	0.25	0%	0%
	0.5	2%	1%
	1	4%	2%
Non-template	0.1	11%	3%
	0.25	20%	3%
	0.5	27%	8%
	1	45%	4%

* The data are presented of n = 4 independent experiments.

* SEM is presented as 95% confidence limit.

Supplementary Table 3.10: Normalized Cy5 intensity of replacement assay (Fig. 6c)

Time (min)	Mean intensity (a.u.)	SEM
0	0.0	0.0
10	1.0	0.0
20	1.8	0.4
30	2.1	0.8
40	3.1	0.6
45	1.8	0.7
50	1.2	0.5

* The data are presented of n = 3 independent experiments.

* SEM is presented as 95% confidence limit.

Supplementary Table 3.11: Fraction of R-loop tested in 1 mM NTP (Fig. 8b)

					P-value in group				*P*-value cross group	
Substrate	Condition	Mean	SEM	N-value	Preformed	NMM	KCl	LiCl	Control	Template
Control	NMM	2%	1%	3						
	KCl	13%	9%	3		0.117797				
	LiCl	0%	-	3						
	No M+	0%	-	3						
Template	Pre-formed G4	17%	4%	3						
	NMM	0%	-	3						
	KCl	9%	7%	3	0.194				0.576	
	LiCl	0%	-	3						
	No M+	0%	-	3						
Non-template	Pre-formed G4	91%	5%	6						1.32E-07
	NMM	55%	8%	5	2.03E-05				1.28E-04	
	KCl	48%	8%	11	2.75E-06	0.413			2.66E-03	1.02E-03
	LiCl	17%	8%	5	3.17E-08	3.83E-04	8.52E-04			
	No M+	12%	5%	3	1.09E-07	5.63E-04	1.56E-03	0.448723		

* N values indicate the number of independent experiments.

* SEM is presented as 95% confidence limit.

* *P*-values are reported by two-sided unpaired t-test.

Supplementary Table 3.12: RNA productions tested in 1 mM NTP (Fig. 8c)

					P-value in group				*P*-value cross group	
Substrate	Condition	Mean	SEM	N-value	Pre-formed G4	NMM	KCl	LiCl	Control	Template
Control	NMM	0.89	0.17	3						
	KCl	1.00	0.17	3		0.231				
	LiCl	0.66	0.20	3		0.101	0.020			
	No M+	0.84	0.16	3		0.647	0.180	0.239		
Template	Pre-formed G4	0.01	0.00	4						
	NMM	0.76	0.09	3	5.04E-05				0.191	
	KCl	0.71	0.10	3	1.56E-05	0.500			7.75E-04	
	LiCl	0.89	0.06	3	2.34E-05	0.319	0.093		0.144	
	No M+	0.60	0.09	3	4.81E-05	0.184	0.288	0.041	0.148	
Non-template	Pre-formed G4	3.47	0.39	4						2.28E-06
	NMM	2.36	0.20	3	1.04E-02				1.48E-03	9.30E-04
	KCl	2.44	0.16	3	8.65E-03	0.701			7.60E-05	4.72E-05
	LiCl	1.69	0.07	3	5.84E-04	0.017	1.34E-03		6.46E-04	3.24E-04
	No M+	1.35	0.20	3	4.02E-04	8.99E-03	1.93E-03	0.082	3.33E-02	6.00E-03

* N values indicate the number of independent experiments.

* RNA productions were normalized to control (KCl).

* SEM is presented as 95% confidence limit.

* *P*-values are reported by two-sided unpaired t-test

Supplementary Table 3.13 RNA productions tested in 1 mM ITP mix (Fig. 8d)

Substrate	Condition	Mean	SEM	N-value	*P*-values in group				*P*-values cross group	
					Pre-formed G4	NMM	KCl	LiCl	Control	Template
Control	NMM	0.84	0.25	3						
	KCl	1.00	0.30	3		0.364				
	LiCl	1.06	0.32	3		0.440	0.829			
	No M	1.15	0.34	3		0.302	0.534	0.756		
Template	Pre-formed G4	0.00	0.00	3						
	NMM	1.02	0.11	3	1.11E-04				0.274	
	KCl	0.89	0.21	3	1.44E-03	0.244			0.333	
	LiCl	0.87	0.16	3	0.003	0.373	0.887		0.569	
	No M	1.00	0.32	3	0.005	0.627	0.703	0.808	0.547	
Non-template	Pre-formed G4	1.24	0.21	3						8.31E-04
	NMM	1.26	0.25	3	0.782				0.238	0.569
	KCl	1.15	0.21	3	0.298	0.527			0.891	0.464
	LiCl	1.10	0.27	3	0.405	0.654	0.827		0.912	0.458
	No M	1.30	0.37	3	0.842	0.956	0.510	0.627	0.795	0.422

* N values indicate the number of independent experiments.

* RNA productions were normalized to control (KCl).

* SEM is presented as 95% confidence limit.

* *P*-values are reported by two-sided unpaired t-test

Supplementary Table 3.14: Fraction of truncated form to NMM intensity (Supplementary Fig. 1)

	NMM intensity		Fraction of truncated form	
PQS Name	Mean	SEM	Mean	SEM
111	1258.4	75.2	89%	6%
222	1096.9	100.7	58%	7%
cMyc	1060.5	99.8	77%	5%
199	1025.0	95.6	48%	12%
177	869.9	66.6	67%	9%
133	751.1	61.9	61%	8%
144	727.8	45.7	59%	8%
155	721.5	71.2	57%	8%
233	670.9	73.6	31%	6%
244	642.1	67.4	34%	6%
255	598.7	59.6	36%	5%
555	541.2	88.7	38%	8%
444	514.2	77.4	28%	6%
333	286.6	35.6	26%	4%
TTA	220.3	30.4	7%	2%
TAA	174.6	14.5	0%	0%
AAA	148.8	15.8	0%	0%
Control	126.2	9.4	0%	0%

* The data are presented of n = 8 independent NMM measurements and n = 4 independent DNA extension assays.

* SEM is presented as 95% confidence limit.

* Pearson’s correlation coefficient is 0.930.

Supplementary Table 3.15: Fraction of non-abortive events (Supplementary Fig. 2e)

				P-value	
Name	Mean	SEM	N value	Control	Template
Control	78%	3%	543		
Template	81%	3%	589	0.2895	
Non-template	83%	3%	551	0.085	0.4866

* The data are represented of n = 3 independent measurements. N value means the total number of samples. SEM is determined by binomial distribution with 95% confidence interval.

* *P*-values are reported by Pearson's chi-squared test (one-sided).

SupplementaryTable 3.16: Fraction of R-looped DNA band (Supplementary Fig. 4d)

	Control		Template		Non-template	
Time (min)	Mean	SEM	Mean	SEM	Mean	SEM
0	0%	0%	0%	0%	0%	0%
10	8%	3%	5%	2%	24%	5%
20	13%	5%	7%	3%	42%	9%
30	15%	4%	8%	3%	45%	8%
40	17%	5%	9%	4%	59%	12%
50	15%	5%	6%	3%	60%	11%
60	18%	6%	8%	3%	53%	5%

* Data are presented of n = 3 independent experiments.

* SEM is presented as 95% confidence limit.

Supplementary Table 3.17: Fraction of 0.1 peak (Supplementary Fig. 4e)

	Control		Template		Non-template	
Time (min)	Mean	SEM	Mean	SEM	Mean	SEM
0	0%	0%	0%	0%	0%	0%
10	34%	4%	30%	5%	44%	7%
20	40%	4%	34%	4%	57%	9%
30	45%	6%	35%	6%	62%	10%
40	46%	5%	33%	6%	67%	8%
50	47%	6%	37%	8%	68%	6%
60	48%	8%	34%	7%	63%	6%

* Data are presented of n = 5 independent experiments.

* SEM is presented as 95% confidence limit.

* Pearson correlation coefficient, r, are 0.97, 0.92, 0.96 for control, template, non-template, respectively.

Supplementary Table 3.18: Fraction of R-loop (Supplementary Fig. 10b)

			P-value	
Name	Mean	SEM	9 bp	16 bp
9bp	62%	12%		
16bp	73%	9%	0.201	
41bp	38%	9%	0.020	7.81E-04

* Data are presented of n = 6 independent experiments.

* SEM is represented as 95% confidence limit.

* *P*-value was reported by two-sided unpaired t-test

Supplementary Table 3.19: Relative RNA production (Supplementary Fig. 10c)

			P-value	
Name	Mean	SEM	9 bp	16 bp
9bp	0.60	0.06		
16bp	1.00	0.00	4.81E-07	
41bp	1.24	0.21	3.48E-04	6.49E-02

* Data are presented of n = 6 independent experiments.

* SEM is represented as 95% confidence limit.

* *P*-value was reported by two-sided unpaired t-test
